# Sliding window constrained fault-tolerant filtering of compressor vibration data

**DOI:** 10.1098/rsos.241957

**Published:** 2025-08-13

**Authors:** Shaolin Hu, Xianxi Chen, Guoxi Sun

**Affiliations:** ^1^ Guangdong Provincial Key Laboratory of Petrochemical Equipment Fault Diagnosis, Guangdong University of Petrochemical Technology, Maoming, People’s Republic of China

**Keywords:** fault-tolerant filtering, random error, outliers, high fidelity, non-smooth sequences

## Abstract

This paper presents a sliding window constrained fault-tolerant filtering method for sampling data in petrochemical instrumentation. The method requires the design of an appropriate sliding window width based on the time series, as well as the expansion of both ends of the series. By utilizing a sliding window constraint function, the method produces a smoothed estimate for the current moment within the window. As the window advances, a series of smoothed estimates of the original sampled data is generated. Subsequently, the original series is subtracted from this smoothed estimate to create a new series that represents the differences between the two. This difference series is then subjected to an additional smoothing estimation process, and the resulting smoothed estimates are employed to compensate for the smoothed estimates of the original sampled series. The experimental results indicate that, compared with sliding mean filtering, sliding median filtering and Savitzky–Golay filtering, the method proposed in this paper can more effectively filter out random errors and reduce the impact of outliers when dealing with sampling data contaminated by noise and outliers. It possesses strong fault tolerance and the ability to extract the true variations of the sampling data.

## 1. Introduction

In the context of petrochemical production, the sampling data obtained from instrumentation are of paramount importance. Specifically, the real-time monitoring of parameters such as temperature, vibration, flow rate and liquid level is essential for optimizing production efficiency and ensuring product quality [1]. However, in practical applications, these sampling data are frequently subject to random errors, resulting in non-smooth time series behaviour. Factors such as equipment noise, disrupted data transmission and sensor malfunctions contribute to the presence of random errors, as well as isolated and patchy outliers within the data. Consequently, this paper aims to investigate effective methodologies for mitigating the impact of outliers and eliminating the interference caused by random errors, thereby facilitating a more accurate representation of the true variations in the instrument sampling subject.

In the field of data processing, filtering and noise reduction techniques are essential for extracting valuable information from complex signals or datasets while minimizing or eliminating noise and interference [2]. In practice, challenges such as sensor malfunctions and transmission interferences often result in the presence of isolated outliers and patchy outliers, which can significantly compromise the efficacy of filtering and noise reduction algorithms [3]. Isolated outliers are defined as singular anomalous values that deviate markedly from the majority of data points [4], whereas patchy outliers are characterized by a localized cluster of consecutively occurring anomalous values that exhibit significant differences from the surrounding data in terms of their attributes [5–7]. Conventional filtering and noise reduction methods, including Winner filtering, Kalman filtering and optimal filters based on frequency domain analysis, typically depend on specific mathematical models and statistical assumptions for their design and application. While these algorithms can perform effectively under ideal conditions, their performance is adversely affected when environmental changes occur, when actual conditions diverge from the mathematical models, or when the data sequence contains outliers [8].

Numerous academic studies have investigated methods for noise reduction filtering and outlier processing in measurement data. For instance, Montenegro *et al.* [9] present a mean filtering algorithm, which effectively mitigates the impact of noise on the signal and reduces signal distortion. However, this approach is limited to signals influenced by Gaussian noise and does not account for scenarios where data are contaminated by outliers. Additionally, Zhu *et al.* [10] propose an outlier rejection method for telemetry data based on Kalman filtering. This method effectively eliminates outliers in the signal while suppressing background noise interference. However, it is important to note that this approach focuses solely on isolated outliers and does not address the issue of patchy outliers. Common strategies for addressing outliers, such as culling or jackknifing [11,12], are analysed in Zhuo [13], which experimentally evaluates existing methods for outliers culling in the context of isolated outliers and patchy outliers. The findings indicate that the methods examined are effective for the rejection of isolated outliers. However, the performance in rejecting patchy outliers is suboptimal, with issues related to the incomplete rejection of outliers persisting. In addition, numerous studies have examined filtering methods that do not require the identification and rejection of outliers. The double median filtering method, as proposed in Hu *et al.* [14], is based on the sliding median filtering technique and demonstrates a commendable fault-tolerance capability. However, despite utilizing the residual sequence from the filtering process for compensation calculations, this method’s effectiveness in retaining information regarding intrinsic changes in the data is constrained, particularly when confronted with complex, non-smooth variations within the window segment. Conversely, the fault-tolerant Q-filtering method introduced in Hu *et al.* [8] employs a quartile-mean operator that calculates the statistical mean of data within the interval defined by the upper and lower quartiles while processing sorted measurement segments and residual segments. This approach is more adept at preserving the true information within the data compared with the double median filtering method. However, neither of these methods expands the first and last values at both ends of the sequence, leading to unavoidable unfiltered original data at both ends of the filtered result. Magar *et al.* [15] established a constraint range by incorporating the interquartile range (IQR). Data points that fall below the lower quartile or exceed the upper quartile by a factor of 1.5 of the IQR are classified as potential outliers. However, in practical applications involving complex, non-smooth sequences, the constraint range may be excessively broad, thereby limiting the method’s effectiveness in addressing outliers, even when they are present.

In response to the aforementioned challenges, this paper presents a sliding window constraint fault-tolerant filtering method for noise reduction. This approach is predicated on the establishment of a constraint range, as previously discussed, and involves the construction of a sliding window constraint function based on quartiles. Constraints are applied to each value within the window to mitigate the impact of outliers on the filtering outcomes while preserving the essential information regarding intrinsic changes. Additionally, the method incorporates the corresponding data sequences at both the initial and final ends of the extension process, thereby preventing the inclusion of unfiltered data at the endpoints of the filtered results. The aforementioned enhancements render the current methodology more broadly applicable and significantly enhance its fault tolerance. This approach effectively filters random errors within the data and mitigates the influence of outliers, and it is capable of processing equally spaced sampling data from various types of both smooth and non-smooth systems. This methodology offers substantial support for the data preprocessing phase in petrochemical industry processes.

## 2. Methods

Data obtained from the petrochemical production process is typically gathered through a multitude of meters [16,17]. For the collected equally spaced data sequences, which are uniformly spaced, analysis is commonly performed using a sliding window approach. In this method, a sliding time window with window width w is constructed for the sequence $S=\left\{y\left(t_{i}\right)|t_{i}=t_{0}+ih,\;i=1,\ldots ,N\right\}$ with a sampling interval of h, encompassing a maximum of w sampling points within the time window. To enhance data processing efficiency, the window width parameter is generally configured to an odd number w=2k+1, and k is an adjustable window radius parameter (preset value k=20).

When the middle position of the window is $t_{i}$, the sample passing through the window contains an exact number of sample points w=2k+1, resulting in the construction of a fragmented representation of the sampled data sequence $\left\{y\left(t_{j}\right)|t_{j}=t_{i}+\left(j-i\right)h,j=i-k,\ldots ,i+k,\;N-k\geq i\geq k+1\right\}\subset S$. This data fragment is then sorted to obtain an ordered sequence $\left\{{\hat{y}}_{j}|j=i-k,\ldots ,i+k\right\}$ from smallest to largest. Three feature points are subsequently extracted: the median $M_{y,i}={\hat{y}}_{i}$, the lower quartile $L_{y,i}={\hat{y}}_{i-[k/2]}$ and the upper quartile $U_{y,i}={\hat{y}}_{i+[k/2]}$, where [ ] denotes the rounding operator.

To mitigate information loss resulting from sequence shortening, both the initial and final segments of the sampled data sequence S are extended by an equivalent value.


(2.1) $$\tilde{S}=\left\{y\left(t_{i}\right)=y\left(t_{1}\right)|i=-k+1,\ldots ,0\right\}\cup S\cup \left\{y\left(t_{i}\right)=y\left(t_{N}\right)|i=N+1,\ldots ,N+k-1\right\}.$$

For the expanded sample sequence $\tilde{S}$, the gradual movement of the window from left to right yields the median sequence $\left\{M_{y,i}|i=1,\ldots ,N\right\}$, as well as the lower quartile sequence $\left\{L_{y,i}|i=1,\ldots ,N\right\}$ and upper quartile sequence $\left\{U_{y,i}|i=1,\ldots ,N\right\}$ derived from $\tilde{S}$.

Informed by the concepts discussed in the preceding section, the constraint ranges are reconstructed utilizing quartiles to mitigate the impact of outliers on the filtering outcomes while preserving valuable information. For the petrochemical instrument sampling data sequence, the sliding window constraint function is developed based on the sequence of three characteristic points: the median, lower quartile and upper quartile


(2.2) $$f_{y}\left(y\left(t_{i+j}\right)\right)=\left\{ \begin{array}{ll} 2U_{y,i}-M_{y,i}, & y\left(t_{i+j}\right)>2U_{y,i}-M_{y,i}\\ y\left(t_{i+j}\right), & 2L_{y,i}\leq y\left(t_{i+j}\right)+M_{y,i}\leq 2U_{y,i}\\ 2L_{y,i}-M_{y,i}, & y\left(t_{i+j}\right)<2L_{y,i}-M_{y,i}. \end{array}\right.$$

Constrained smoothing of the sample data within a specified window is conducted using equation (2.2), resulting in window constrained smoothing estimate at a given time $t_{i}$:


(2.3) $$\hat{y}\left(t_{i}\right)=\frac{1}{2k+1}\sum_{j=-k}^{k}f_{y}\left(y\left(t_{i+j}\right)\right)\;\left(i=1,\ldots ,N\right).$$

To mitigate the negative impact of constrained smoothing on the trending component of the sampled data, a series of smoothed filtered residuals is generated $E=\left\{\varepsilon \left(t_{i}\right)=y\left(t_{i}\right)-\hat{y}\left(t_{i}\right)|i=1,\ldots ,N\right\}$. Concurrently, to avert the loss of information resulting from the truncation of the sequence, a zero-value expansion is implemented at both the beginning and the end of the sequence of smoothed filtered residuals E:


(2.4) $$\tilde{E}=\left\{\varepsilon \left(t_{i}\right)=0|i=-k+1,\ldots ,0\right\}\cup E\cup \left\{\varepsilon \left(t_{i}\right)=0|i=N+1,\ldots ,N+k-1\right\}.$$

To mitigate the influence of outliers on the estimation of residual smoothing, it is necessary to implement constrained smoothing of the residual sequences. For the expanded residual sequence $\tilde{E}$, the window is progressively shifted from left to right, resulting in the generation of the median sequence$\;\left\{M_{\varepsilon ,i}|i=1,\ldots ,N\right\}$, as well as the lower-quartile sequence $\left\{L_{\varepsilon ,i}|i=1,\ldots ,N\right\}$and upper-quartile sequence $\left\{U_{\varepsilon ,i}|i=1,\ldots ,N\right\}$. Consequently, a sliding-window residual constraint function is established.


(2.5) $$f_{\varepsilon }\left(\varepsilon \left(t_{i+j}\right)\right)=\left\{ \begin{array}{ll} 2U_{\varepsilon ,i}-M_{\varepsilon ,i}, & \varepsilon \left(t_{i+j}\right)>2U_{\varepsilon ,i}-M_{\varepsilon ,i}\\ \varepsilon \left(t_{i+j}\right), & 2L_{\varepsilon ,i}\leq \varepsilon \left(t_{i+j}\right)+M_{\varepsilon ,i}\leq 2U_{\varepsilon ,i}\\ 2L_{\varepsilon ,i}-M_{\varepsilon ,i}, & \varepsilon \left(t_{i+j}\right)<2L_{\varepsilon ,i}-M_{\varepsilon ,i}. \end{array}\right.$$

Constrained smoothing of the sample data within a specified window is conducted using equation (2.5), resulting in window constrained smoothing estimate at a given time $t_{i}$:


(2.6) $$\hat{\varepsilon }\left(t_{i}\right)=\frac{1}{2k+1}\sum_{j=-k}^{k}f_{\varepsilon }\left(\varepsilon \left(t_{i+j}\right)\right)\;\left(i=1,\ldots ,N\right).$$

The compensation of the primary smoothing filter sequence $\left\{\hat{y}\left(t_{i}\right)|i=1,\ldots ,N\right\}$ is conducted in a superimposed manner utilizing residual smoothing estimation $\left\{\hat{\varepsilon }\left(t_{i}\right)|i=1,\ldots ,N\right\}$


(2.7) $$\tilde{y}\left(t_{i}\right)=\hat{y}\left(t_{i}\right)+\hat{\varepsilon }\left(t_{i}\right)\;\left(i=1,\ldots ,N\right).$$

**Figure 1.**
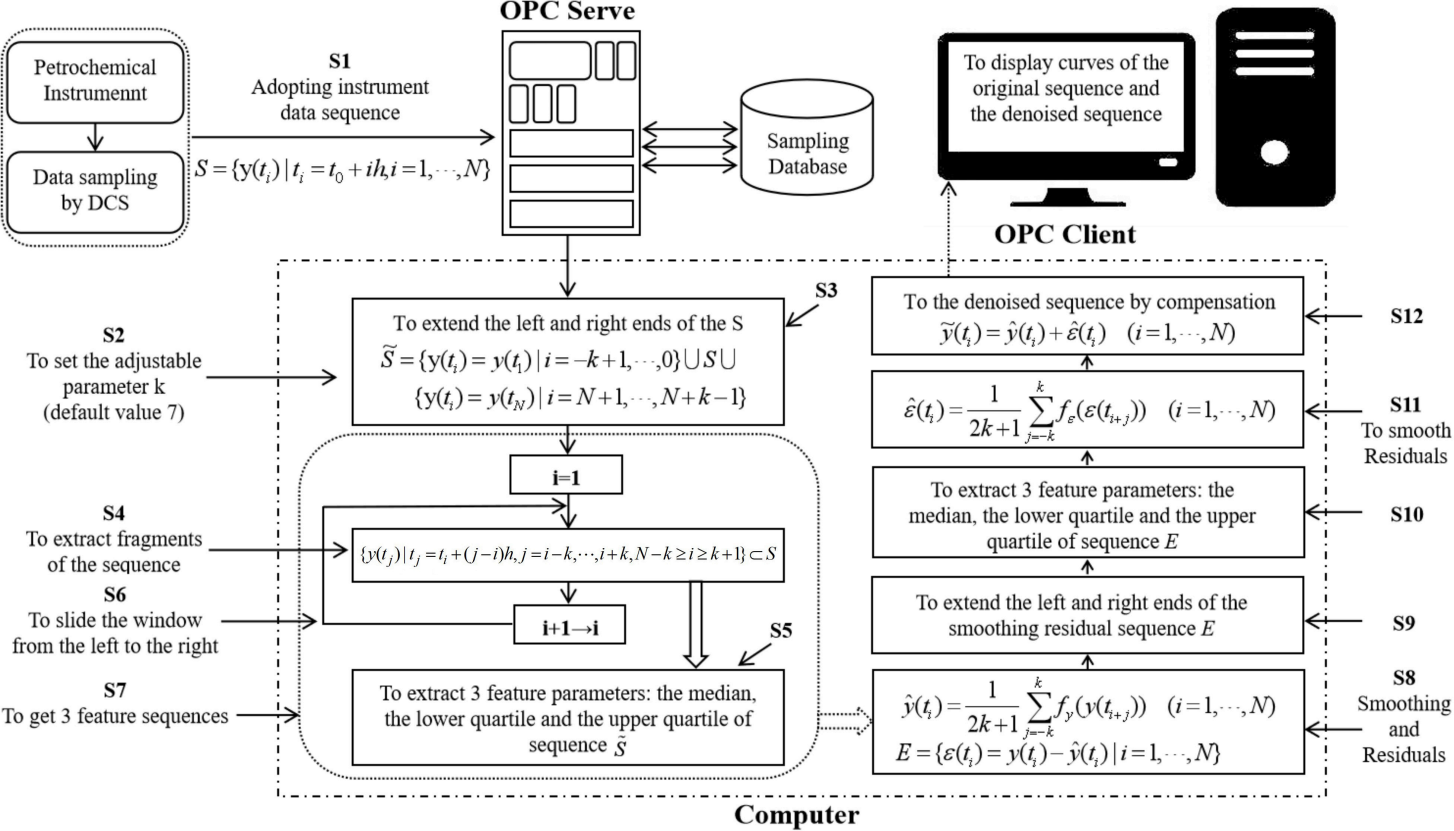
Flowchart of sliding window constrained fault-tolerant filtering method.

The results of filtering the petrochemical instrumentation sampling data $S=\left\{y\left(t_{i}\right)|t_{i}=t_{0}+ih,\;i=1,\ldots ,N\right\}$ have been obtained. The flowchart illustrating the methodology employed in this study is presented in figure 1.

## 3. Performance metrics for algorithms

To assess the effectiveness of filtering, it is essential to establish performance metrics for analysis. The primary function of filtering is to mitigate the impact of random errors while simultaneously preserving the genuine changes in the data. Consequently, the evaluation of filtering efficacy involves the introduction of fault tolerance, the degree of deformation of the curve under various influences, the localized difference of curve fluctuations and an analysis of the random errors present in the filter residuals.

### 3.1. Fault tolerance and the degree of deformation of the curve under influence

The fault tolerance of a filtering method is typically characterized by its capacity to remain unaffected by outliers when the data are contaminated by such anomalies. This paper introduces the concept of the sample breakdown point for analytical purposes, which is defined as smallest percentage of outliers in the sample that maximizes the maximum absolute deviation of the smoothed estimate. Higher breakdown point indicates a more stable estimate, although it should not exceed 50% [18]. For the filtering algorithm ${\tilde{y}}_{i}=\chi (y_{1},\ldots ,y_{m})$, we define [19]


(3.1) $$b\left(\frac{r}{m}\right)=\max_{i_{1},\ldots ,i_{r}}\underset{y_{i_{1}}^{*},\cdots ,y_{i_{r}}^{*}}{\sup}\left\{∥\chi \left(y_{1},\ldots ,y_{i_{1}}^{*},\ldots ,y_{i_{r}}^{*},\ldots ,y_{m}\right)-\chi \left(y_{1},\ldots ,y_{i_{1}},\ldots ,y_{i_{r}}\ldots ,y_{m}\right)∥\right\}.$$

The sample breakdown point is subsequently expressed as


(3.2) $$\varepsilon \left(\chi \right)=\frac{1}{m}\min\left\{r:b\left(\frac{r}{m}\right)=b\left(1\right)\right\}.$$

In equation (3.1), $y(t_{i})$ is denoted simply as $y_{i}$. In this context, r represents the quantity of contaminated data, m signifies the total amount of data and $y_{i_{1}}^{*},\ldots ,y_{i_{r}}^{*}$refers to the contaminated data.

Secondly, during the stage of algorithm performance analysis and evaluation using simulated or measured data, the absence of outlier samples in the data makes it difficult to directly assess the algorithm’s robustness against outliers. To more intuitively demonstrate the algorithm’s ability to resist outliers, randomly generated outlier points are added to the data to be processed. Additionally, to more specifically and intuitively determine the impact of outliers on filtering performance, a performance metric defining the degree of curve deformation affected by outliers is established: the original dataset is denoted as $y(t_{i})$, while the dataset that includes outliers is represented as $\overset{˘}{y}(t_{i})$. Both datasets are subjected to separate filtering processes, resulting in $\tilde{\tilde{y}}(t_{i})$, which is derived from the filtering of the outlier-free data, and $\tilde{y}(t_{i})$, which is obtained from the filtering of the data with outliers. The difference between $\tilde{y}(t_{i})$ and $\tilde{\tilde{y}}(t_{i})$ is calculated, and the absolute values of this difference are taken. Subsequently, the mean of these absolute values is defined as the performance metric for assessing the degree of deformation of the curve under influence (DDCI):


(3.3) $$\text{DDCI}=\frac{\sum_{i=1}^{N}\left|\tilde{y}\left(t_{i}\right)-\tilde{\tilde{y}}\left(t_{i}\right)\right|}{N}.$$

If the DDCI value is larger, it indicates that the filtered results of data containing outliers differ significantly from the filtered results of data without outliers. The curve deformation is pronounced, which means that the filtering method cannot effectively eliminate the influence of outliers. Conversely, when DDCI is smaller, the filtered results of data containing outliers are similar to those of data without outliers, with minimal curve deformation. This suggests that the filtering method can effectively eliminate the influence of outliers, prevent numerical anomalies in the filtering results and better restore the true variations of the data.

### 3.2. Local fluctuation difference of the curves

In the context of local abnormal fluctuations within a curve, particularly those arising from local data deformations induced by certain filtering methods in regions of non-monotonic data variation, as well as the presence of irregularities such as burrs, the concept of first-order differences is employed to analyse the variations between adjacent points on the data curve [20]. This approach facilitates the assessment of the degree of local abnormal fluctuations present in the curve. In the analysis, the first-order differences of every two adjacent points in the resultant vector $\tilde{y}(t_{i})$, obtained after filtering, are computed. The absolute values of these differences are then divided by the sampling interval to generate a sequence of differences between adjacent points. Subsequently, the standard deviation of this sequence is calculated. This process defines the performance metrics for assessing the local fluctuation difference of the curve (LFDC).


(3.4) $$\text{LFDC}=\text{std}\left\{\frac{\left|\tilde{y}\left(t_{i+1}\right)-\tilde{y}\left(t_{i}\right)\right|}{h}\;|\;i=1,\ldots ,N-1\right\}.$$

In this context, h represents the sampling interval, while std( ) denotes the standard deviation operator. A small standard deviation indicates that the variations between adjacent points in the time series are consistent across different time points, suggesting that the changes observed at each adjacent point are largely similar. If the standard deviation is large, it indicates that there is a significant difference in the change between adjacent data points in certain local areas compared with the changes at other time points. This suggests that there may be pronounced local data deformation, distortion or a lack of smoothness in the curve.

### 3.3. Random error analysis of filtered residuals

**Figure 2.**
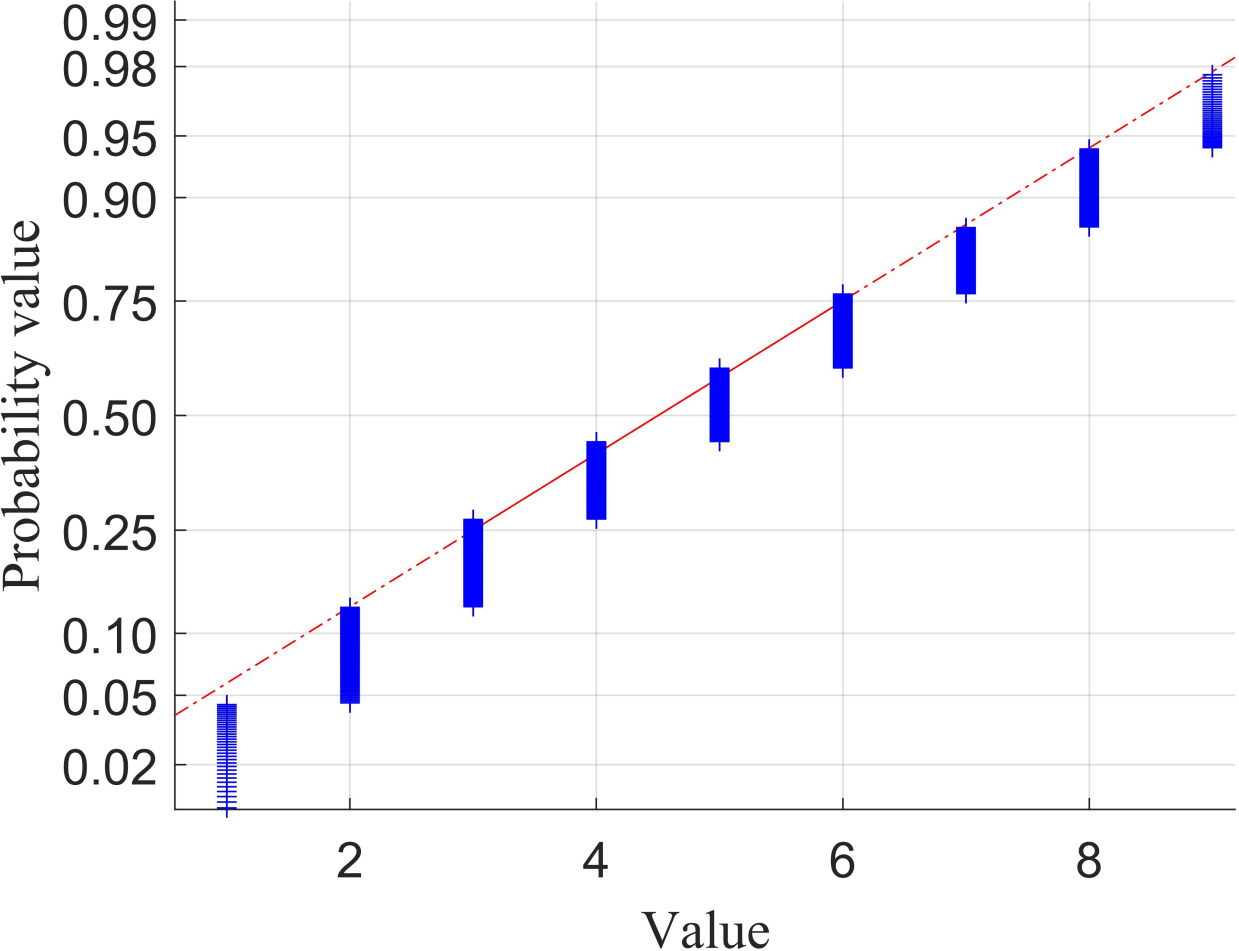
Normal probability plots (using randomly generated non-normally distributed data).

The majority of random error distribution laws conform to a normal distribution [21]. Consequently, this paper primarily examines the filter residuals in relation to their adherence to normal distribution and their distribution characteristics. To facilitate this analysis, a normal probability plot is constructed to compare the empirical data distribution with the theoretical normal distribution. Each data point is represented by a ‘+’ symbol, and two reference lines indicative of the theoretical distribution are included; the solid reference line connects the first and third quartiles of the data, while the dashed reference line extends the solid line to encompass the entirety of the data. If the sample data exhibit a normal distribution, the data points will align along the reference line. In the normal probability plot, the horizontal axis represents the variable interval, whereas the vertical axis denotes the quartiles of the normal distribution, which are converted into probability values. As illustrated in figure 2, the data presented in this figure consist of randomly generated data that do not conform to a normal distribution.

When the data points are situated closer to the reference line, it indicates a higher degree of conformity to the normal distribution. This suggests that the sample largely adheres to the assumption of normality. In other words, a greater alignment of the filtered residuals with random errors implies that this filtering method is more effective in isolating random errors within the dataset while simultaneously preserving the non-random components that hold practical significance. Conversely, if a substantial number of sample points are not positioned near the reference line, it suggests that the filtered residuals exhibit a lesser degree of adherence to the characteristics of a normal distribution. In such cases, those points that deviate significantly cannot be regarded as samples conforming to a normal distribution.

The aforementioned three metrics provide a more comprehensive reflection and evaluation of the level of fault tolerance and the effectiveness of the filtering process.

## 4. Results

To intuitively illustrate the filtering effect of the sliding window constrained fault-tolerant filtering method and to highlight its superior fault-tolerant performance, this paper conducts a comparative analysis of sliding median filtering [22], sliding mean filtering [23] and Savitzky–Golay filtering [24], all of which are widely utilized in the engineering domain. The methods, including the one proposed in this paper, are applied to both simulation data containing outliers and actual data, facilitating a comprehensive analysis and comparison.

### 4.1. Simulation analysis

In this section, wave signals and standard polynomial signals that incorporate varying trend components, periodic components, and perturbation components are utilized as simulation data. Model (1) and (2) have been selected for this analysis.


$$Model\;(1):\;y_{1}\left(t_{i}\right)=-15t_{i}+20\sin\left(10\pi t_{i}\right)+2\mu \left(t_{i}\right).$$

$$Model\;(2):\;y_{2}\left(t_{i}\right)=12t_{i}\left(10t_{i}+1\right).$$

Generate 500 simulation data points at 1 s intervals and 800 simulation data points at 1 s intervals, respectively, where $\mu \left(t_{i}\right)∼N\left(0,1\right)$. Additionally, incorporate outlier processing in the wave signal simulation data.


(4.1) $$\begin{array}{rl} \left\{{\overset{˘}{y}}_{1}(t_{i}\right) & =y_{1}(t_{i})-80|i=100\}\\ \left\{{\overset{˘}{y}}_{1}(t_{i}\right) & =y_{1}(t_{i})+80|i=190,\ldots ,192\}\\ \left\{{\overset{˘}{y}}_{1}(t_{i}\right) & =y_{1}(t_{i})-80|i=300\}\\ \left\{{\overset{˘}{y}}_{1}(t_{i}\right) & =y_{1}(t_{i})+70|i=408,\ldots ,416\}. \end{array}$$

The processing of additive outliers in polynomial signal simulation data.


(4.2) $$\begin{array}{rl} \left\{{\overset{˘}{y}}_{2}(t_{i}\right) & =y_{2}(t_{i})+90|i=100\}\\ \left\{{\overset{˘}{y}}_{2}(t_{i}\right) & =y_{2}(t_{i})+75|i=270,\ldots ,278\}\\ \left\{{\overset{˘}{y}}_{2}(t_{i}\right) & =y_{2}(t_{i})-50|i=420,\ldots ,428\}\\ \left\{{\overset{˘}{y}}_{2}(t_{i}\right) & =y_{2}(t_{i})-100|i=660\}. \end{array}$$

**Figure 3.**
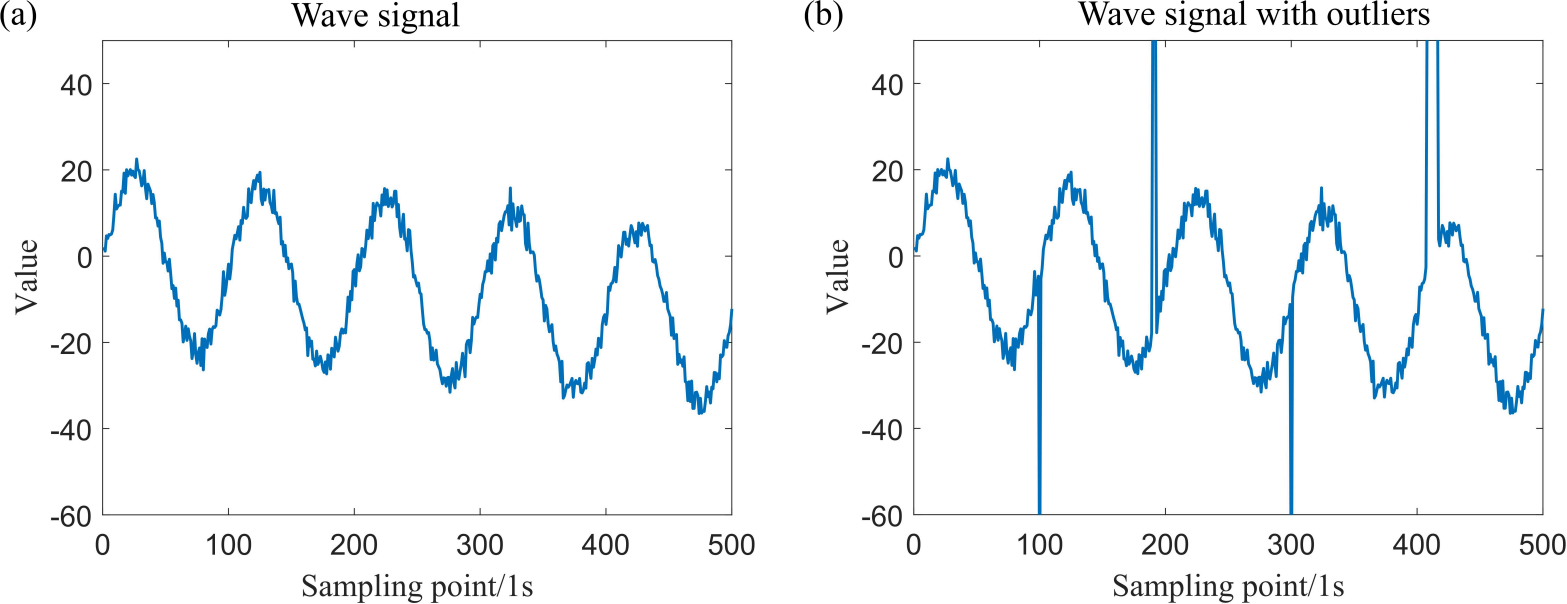
Simulation data generated by Model (1).

**Figure 4.**
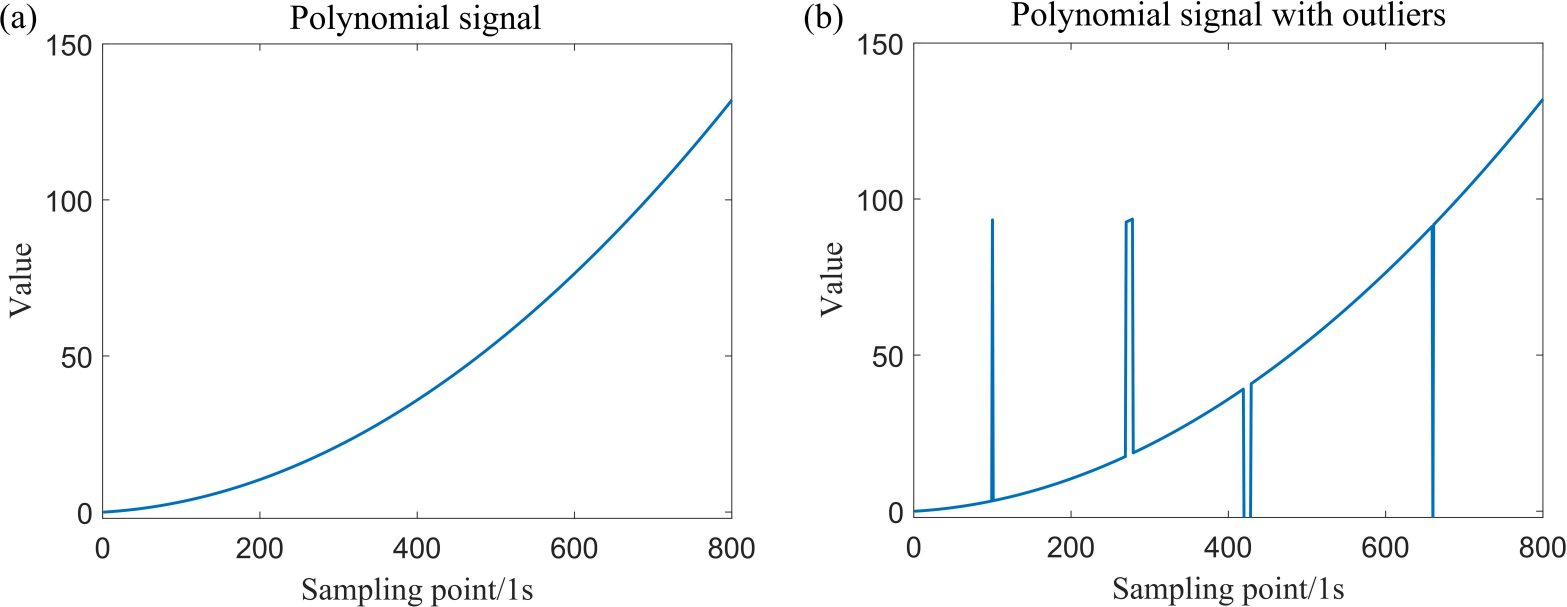
Simulation data generated by Model (2).

The simulation data for a total of four locations, which include isolated outliers and patchy outliers, have been generated. The original simulation data, along with the simulation data subsequent to the introduction of outliers, are presented in figures 3 and 4.

**Figure 5.**
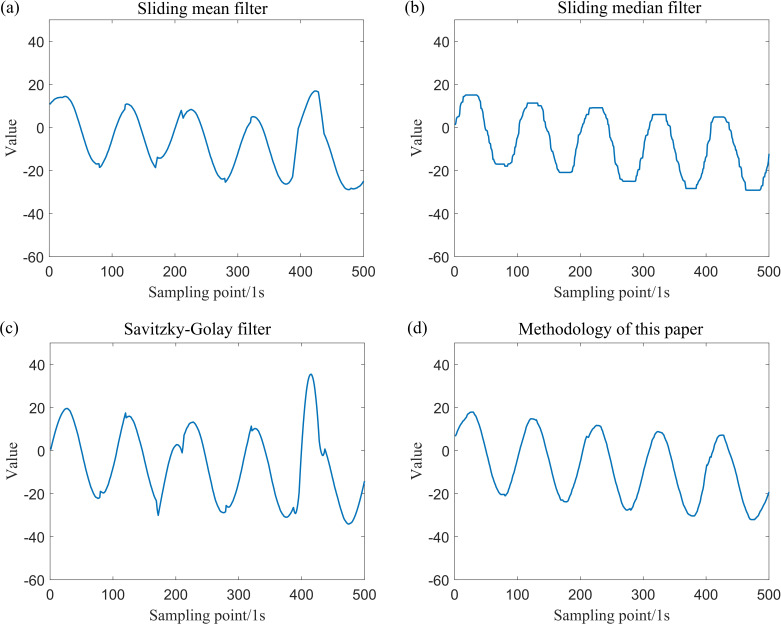
Comparison of the effect of wave signal after using four filtering methods.

**Figure 6.**
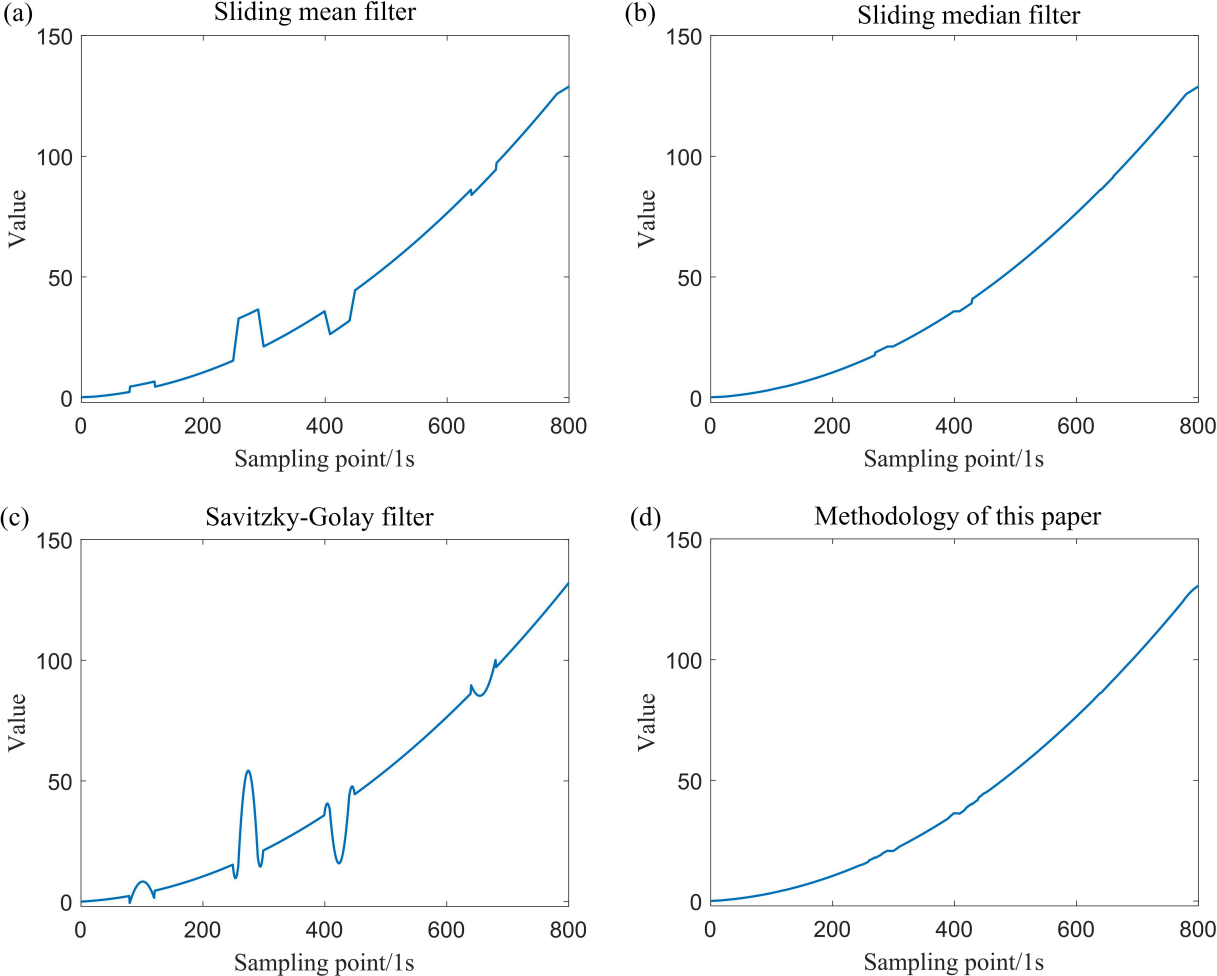
Comparison of the effects of polynomial signal after using four filtering methods.

After incorporating outliers into the simulation data generated by Model (1) and Model (2), the data were processed using various filtering techniques, including the sliding mean filter, sliding median filter, Savitzky–Golay filter and the sliding window constrained fault-tolerant filtering method. The parameters for the sliding window radius were set to k=20. The results of these filtering methods are presented in figures 5 and 6.

**Figure 7.**
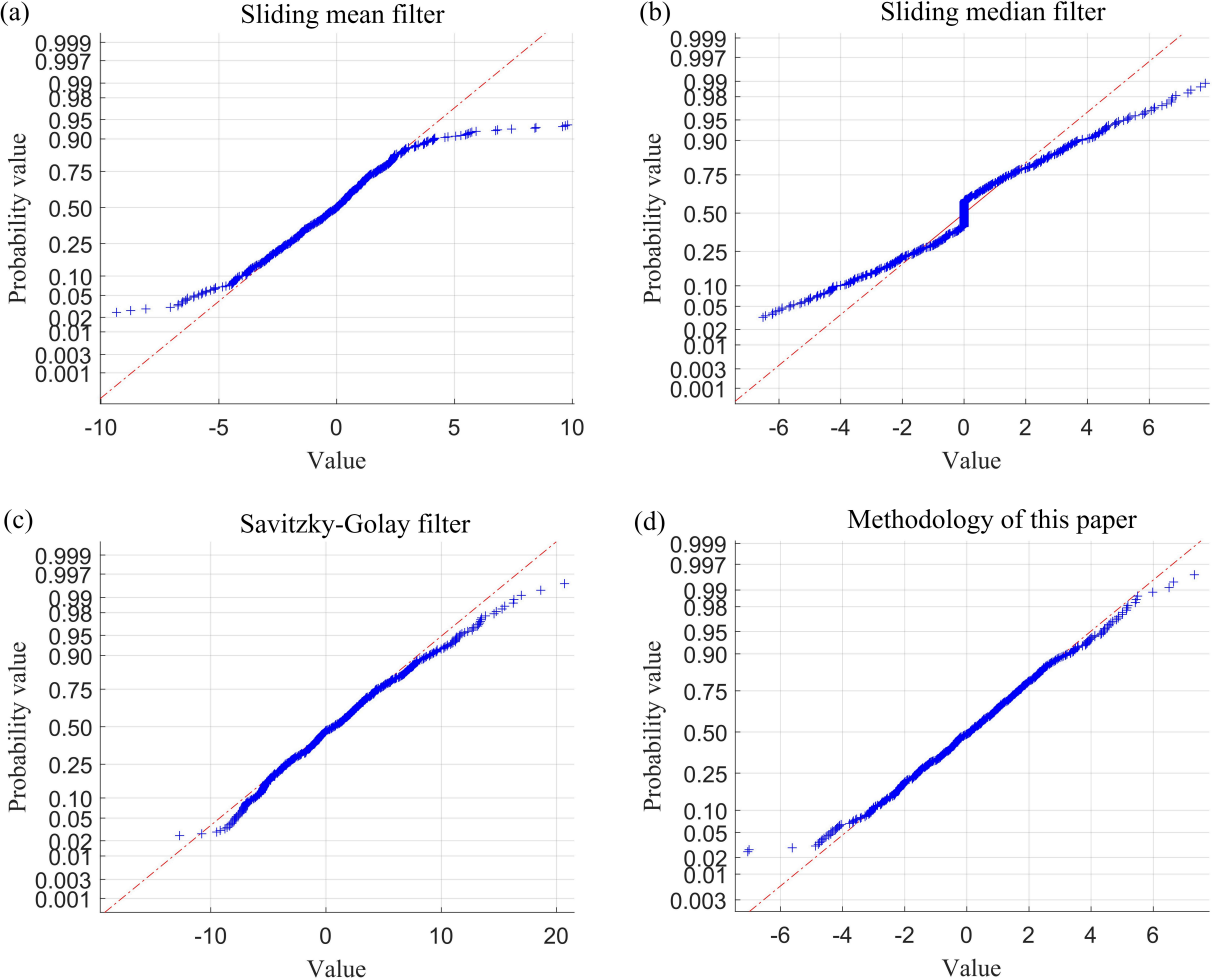
Normal probability plot of filtered residuals using four filtering methods for wave signal after adding outliers.

The simulation outcomes are analysed in terms of fault-tolerance capability, the degree of curve deformation, and the difference of local fluctuations in the performance metrics of the curve, and random error analysis of the filter residuals. The performance metrics obtained in this section are summarized in table 1 and figure 7.

**Table 1.** Performance evaluation of filtering effects on simulated data. Bold values indicate statistical significance.

filtering method	wave signal		polynomial signal	
	DDCI	LFDC	DDCI	LFDC
sliding mean filter	2.0600	0.4744	1.6438	0.3376
sliding median filter	0.3427	0.9524	0.0903	0.1104
Savitzky–Golay filter	2.6090	0.7770	2.0491	0.6641
methodology of this paper	**0.2477**	**0.4320**	**0.0721**	**0.0924**

From the definition provided in [14], it is evident that the sample breakdown point of the sliding mean filter and the Savitzky–Golay filter is 0, while the sample breakdown point of the sliding median filter is 1/2. By contrast, the sample breakdown point of the method proposed in this paper is approximately 1/4. The following section offers a clearer understanding of the sample breakdown points through visualization. An analysis of figures 3–7 and table 1 indicates that both the sliding mean filter and the Savitzky–Golay filter can smooth data to a certain extent. However, when confronted with data that contain isolated outliers and patchy outliers, the analysis of sample breakdown points reveals that the presence of outliers triggers a deformation in the curve shape. Furthermore, when processing polynomial signals, the performance of the sliding mean filter and the Savitzky–Golay filter is inferior to that of the other two methods across all performance metrics. By contrast, the sliding median filter and the method proposed in this paper effectively mitigate the interference caused by isolated outlier and patchy outliers. Although the sample breakdown point of the sliding median filter is 1/2, the degree of curve deformation observed when processing wave signals and polynomial signals is greater than that of the method presented in this paper. From figures 5 and 7, it is evident that the sliding median filtering method removes a portion of the intrinsic variation information when processing the wave signal. Consequently, the filtered residual sample points exhibit significant deviation from the reference line. In other words, this filtering method eliminates the non-random components of the data that hold practical significance. Consequently, the performance metric for local fluctuation difference of the curve is inferior to that of the method proposed in this paper, with significant discrepancies observed between the change of some adjacent points compared with other time points, leading to the occurrence of deformation phenomena. Therefore, the difference of local fluctuations in the curves produced by the method proposed in this paper is considerably lower, and its sample breakdown point being close to 1/4. This characteristic endows the method with a robust fault-tolerance capability, resulting in minimal deformation of the curve and ensuring the stability of the filtering process.

### 4.2. Practical applications

According to the measured vibration data from the drive-end shaft and exciter shaft of a gas pipeline compressor, the number of data sampling points is *n* = 2000, with a sampling interval of 60 s. Due to the absence of outlier samples in the measured data, three sets of outlier points are randomly generated to more intuitively demonstrate the algorithm’s resistance to outliers. The measured vibration data of the drive-end shaft are now processed by adding outliers. In table 2, the ‘1’ in variable ${{\overset{˘}{y}}^{1}}_{I}(t_{i})$ represents the drive-end shaft vibration data, while ‘*I*’ denotes the first instance of the outlier position, and so forth.

**Table 2.** Addition of outliers in drive-end shaft vibration data.

case no.	drive end shaft vibration data
case I:	$\left\{{\overset{˘}{y}}_{I}^{1}\left(t_{i}\right)=y\left(t_{i}\right)+17|i=200\right\}$ $\left\{{\overset{˘}{y}}_{I}^{1}\left(t_{i}\right)=y\left(t_{i}\right)-5|i=600,\ldots ,608\right\}$ $\left\{{\overset{˘}{y}}_{I}^{1}\left(t_{i}\right)=y\left(t_{i}\right)-6|i=1200,\ldots ,1208\right\}$ $\left\{{\overset{˘}{y}}_{I}^{1}\left(t_{i}\right)=y\left(t_{i}\right)+16|i=1600\right\}$ $\left\{{\overset{˘}{y}}_{I}^{1}\left(t_{i}\right)=y\left(t_{i}\right)+15|i=1800,\ldots ,1808\right\}$
case II:	$\left\{{\overset{˘}{y}}_{II}^{1}\left(t_{i}\right)=y\left(t_{i}\right)-13|i=300,\ldots ,308\right\}$ $\left\{{\overset{˘}{y}}_{II}^{1}\left(t_{i}\right)=y\left(t_{i}\right)+10|i=750\right\}$ $\left\{{\overset{˘}{y}}_{II}^{1}\left(t_{i}\right)=y\left(t_{i}\right)+16|i=1330\right\}$ $\left\{{\overset{˘}{y}}_{II}^{1}\left(t_{i}\right)=y\left(t_{i}\right)-10|i=1718\right\}$ $\left\{{\overset{˘}{y}}_{II}^{1}\left(t_{i}\right)=y\left(t_{i}\right)-13|i=1900,\ldots ,1905\right\}$
case III:	$\left\{{\overset{˘}{y}}_{III}^{1}\left(t_{i}\right)=y\left(t_{i}\right)-20|i=210,\ldots ,213\right\}$ $\left\{{\overset{˘}{y}}_{III}^{1}\left(t_{i}\right)=y\left(t_{i}\right)-11|i=640,\ldots ,642\right\}$ $\left\{{\overset{˘}{y}}_{III}^{1}\left(t_{i}\right)=y\left(t_{i}\right)+14|i=1260\right\}$ $\left\{{\overset{˘}{y}}_{III}^{1}\left(t_{i}\right)=y\left(t_{i}\right)-13|i=1680,\ldots ,1683\right\}$ $\left\{{\overset{˘}{y}}_{III}^{1}\left(t_{i}\right)=y\left(t_{i}\right)-9|i=1860,\ldots ,1868\right\}$

**Figure 8.**
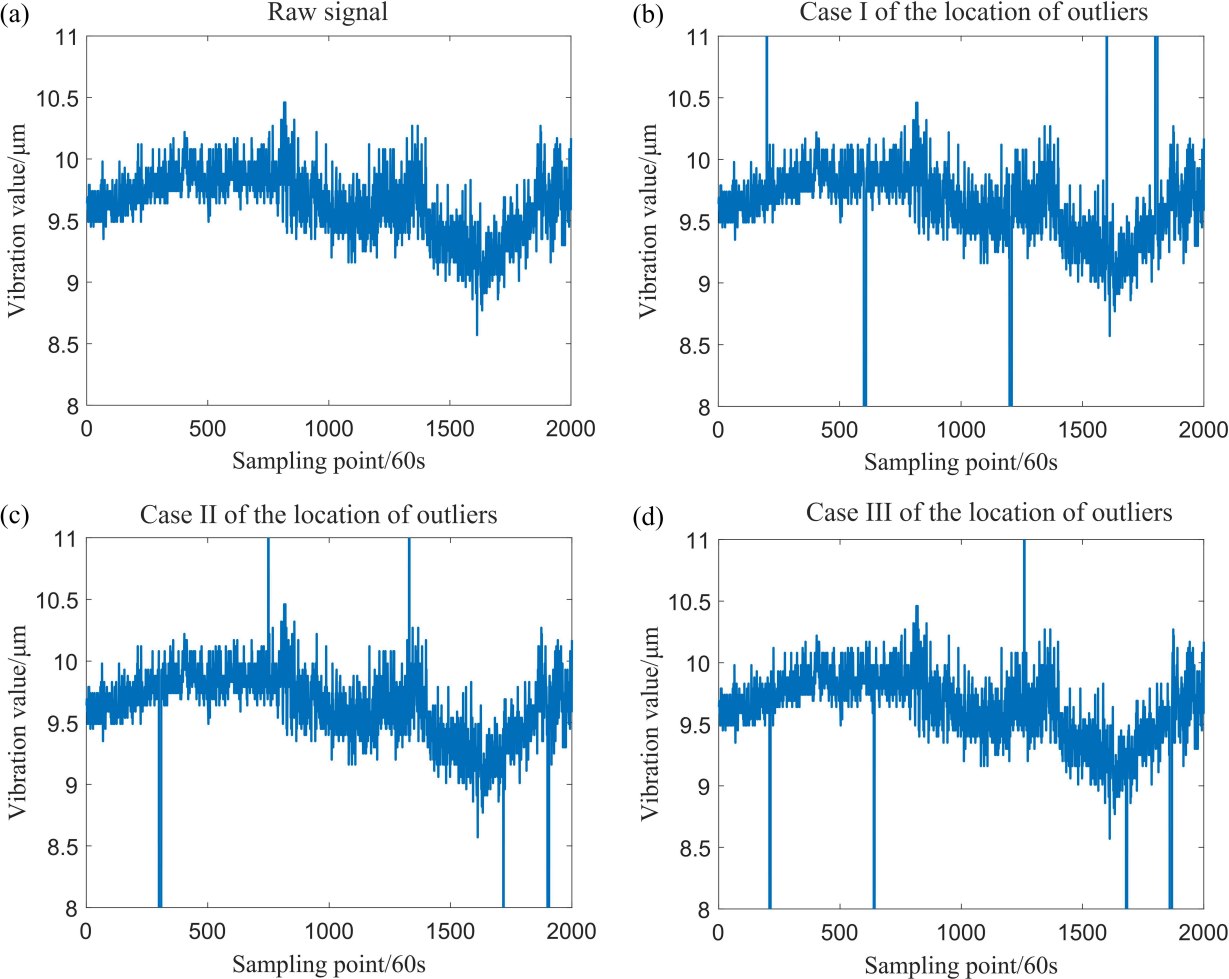
The drive-end shaft vibration data and subsequently added outliers.

Sample data were generated, including patchy outliers with a maximum patch length of 9, patchy outliers of varying lengths and some isolated outliers. The drive-end shaft vibration data, along with the data after the addition of outliers, are presented in figure 8.

**Figure 9.**
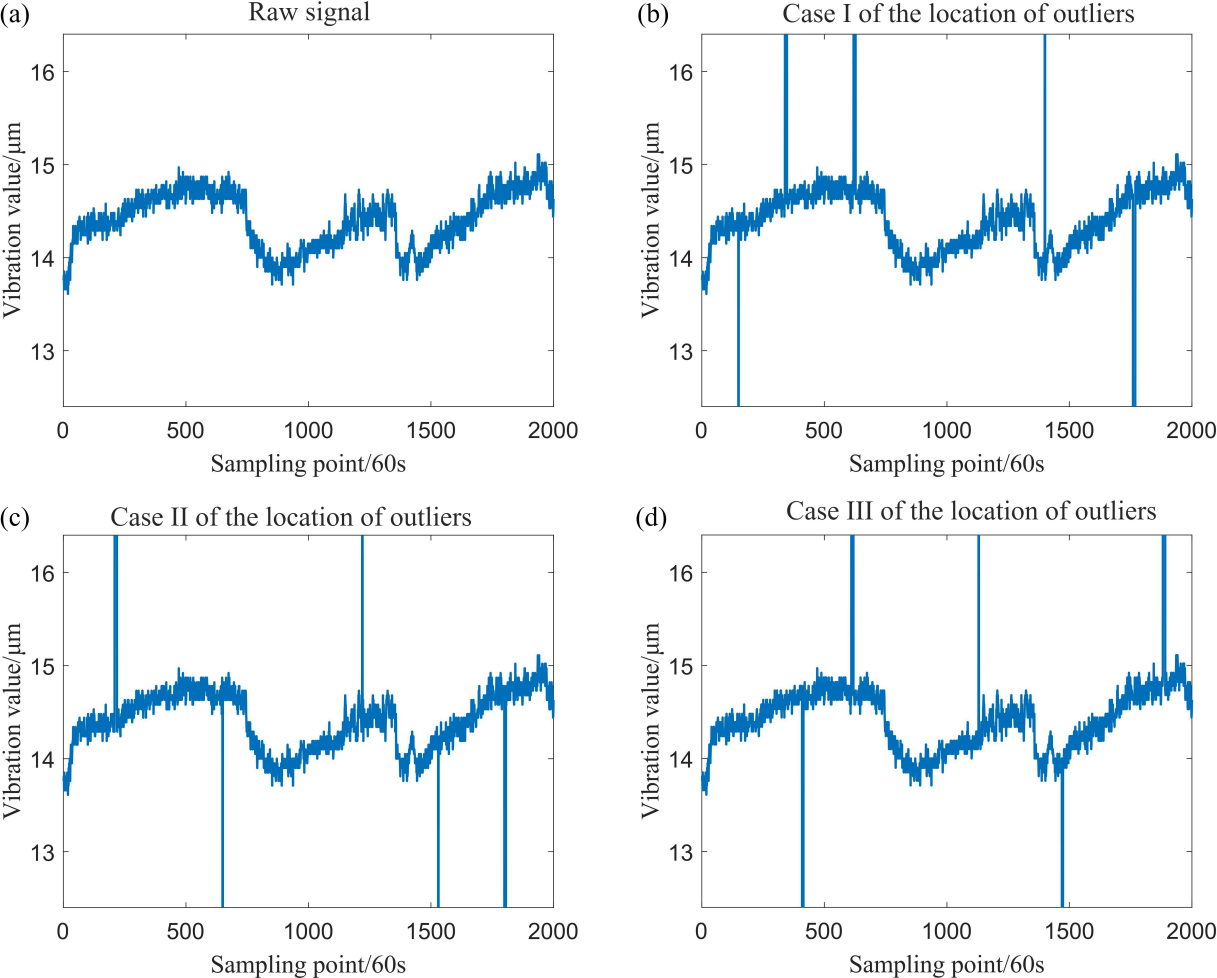
The exciter shaft vibration data and subsequently added outliers.

Similarly, the measured data of exciter shaft vibration are subjected to the addition of outliers, as described above. Three sets of outlier points are randomly generated, and the results of incorporating these outliers into the measured data of exciter shaft vibration are presented in figure 9. In table 3, the ‘2’ in variable ${{\overset{˘}{y}}^{2}}_{I}(t_{i})$ represents the exciter shaft vibration data, while ‘*I*’ denotes the first instance of the outlier position, and so forth.

**Table 3.** Addition of outliers in exciter shaft vibration data.

case no.	exciter shaft vibration data
case I:	$\left\{{\overset{˘}{y}}_{I}^{2}\left(t_{i}\right)=y\left(t_{i}\right)-12|i=150\right\}$ $\left\{{\overset{˘}{y}}_{I}^{2}\left(t_{i}\right)=y\left(t_{i}\right)+6|i=340,\ldots ,347\right\}$ $\left\{{\overset{˘}{y}}_{I}^{2}\left(t_{i}\right)=y\left(t_{i}\right)+7|i=620,\ldots ,627\right\}$ $\left\{{\overset{˘}{y}}_{I}^{2}\left(t_{i}\right)=y\left(t_{i}\right)+17|i=1400\right\}$ $\left\{{\overset{˘}{y}}_{I}^{2}\left(t_{i}\right)=y\left(t_{i}\right)-10|i=1760,\ldots ,1768\right\}$
case II:	$\left\{{\overset{˘}{y}}_{II}^{2}\left(t_{i}\right)=y\left(t_{i}\right)+11|i=210,\ldots ,218\right\}$ $\left\{{\overset{˘}{y}}_{II}^{2}\left(t_{i}\right)=y\left(t_{i}\right)-10|i=650\right\}$ $\left\{{\overset{˘}{y}}_{II}^{2}\left(t_{i}\right)=y\left(t_{i}\right)+16|i=1220\right\}$ $\left\{{\overset{˘}{y}}_{II}^{2}\left(t_{i}\right)=y\left(t_{i}\right)-12|i=1530\right\}$ $\left\{{\overset{˘}{y}}_{II}^{2}\left(t_{i}\right)=y\left(t_{i}\right)-10|i=1800,\ldots ,1805\right\}$
case III:	$\left\{{\overset{˘}{y}}_{III}^{2}\left(t_{i}\right)=y\left(t_{i}\right)-15|i=410,\ldots ,414\right\}$ $\left\{{\overset{˘}{y}}_{III}^{2}\left(t_{i}\right)=y\left(t_{i}\right)+9|i=611,\ldots ,618\right\}$ $\left\{{\overset{˘}{y}}_{III}^{2}\left(t_{i}\right)=y\left(t_{i}\right)+11|i=1130\right\}$ $\left\{{\overset{˘}{y}}_{III}^{2}\left(t_{i}\right)=y\left(t_{i}\right)-13|i=1470,\ldots ,1473\right\}$ $\left\{{\overset{˘}{y}}_{III}^{2}\left(t_{i}\right)=y\left(t_{i}\right)+10|i=1882,\ldots ,1890\right\}$

**Figure 10.**
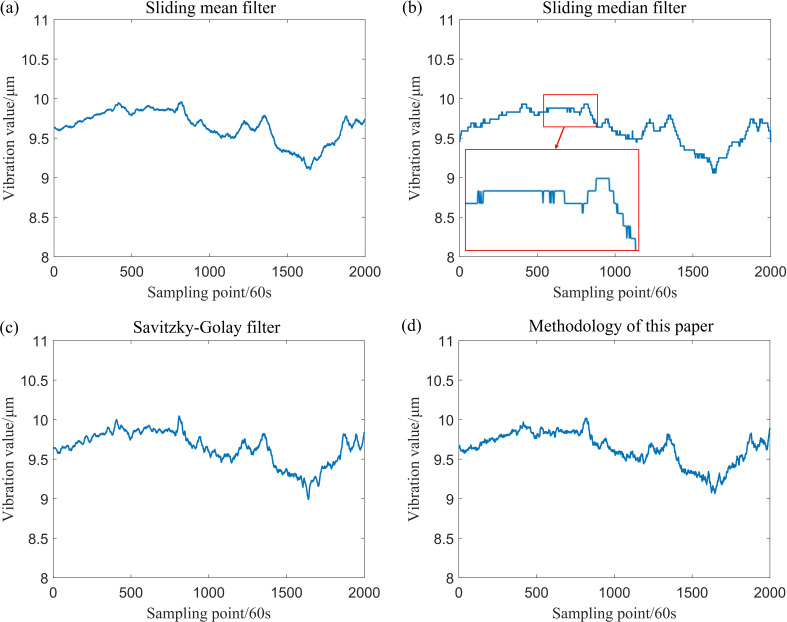
Comparative analysis of the drive-end shaft vibration data utilizing four filtering methods.

**Figure 11.**
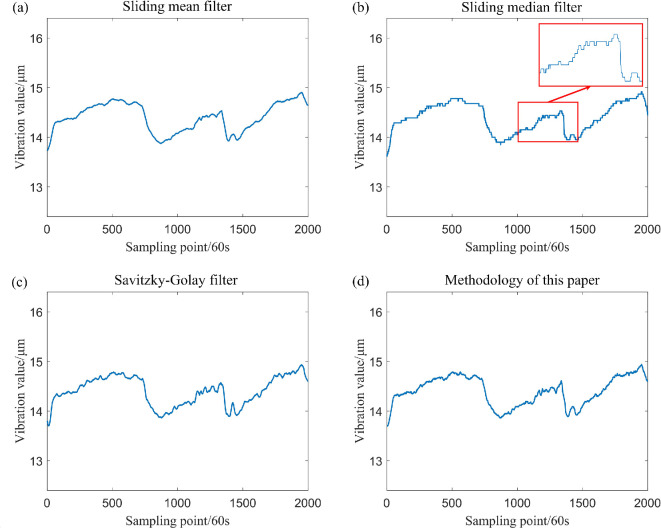
Comparative analysis of the exciter shaft vibration data utilizing four filtering methods.

The sliding window parameter is set to k=20. In the filtering process, the sliding mean filter, sliding median filter, Savitzky–Golay filter and the filtering method proposed in this study are employed (as illustrated in figures 10 and 11). When sampling without outliers or anomalies, the root mean square errors (RMSE) of the four filtering methods are presented in table 4 .

**Table 4.** Root mean square errors (RMSE) of the four filtering methods.

filtering method	drive-end shaft vibration data	exciter shaft vibration data
	RMSE	RMSE
sliding mean filter	0.1573	0.0855
sliding median filter	0.1591	0.0860
Savitzky–Golay filter	0.1561	0.0790
methodology of this paper	0.1606	0.0859

The statistical significance and filtering efficacy of these four methods are comparable when no outliers are present in the data. Furthermore, a comparison of figures 10 and 11 indicates that the sliding median filtering method removes a portion of the data that contains genuine variations, resulting in a distortion of the data.

**Figure 12.**
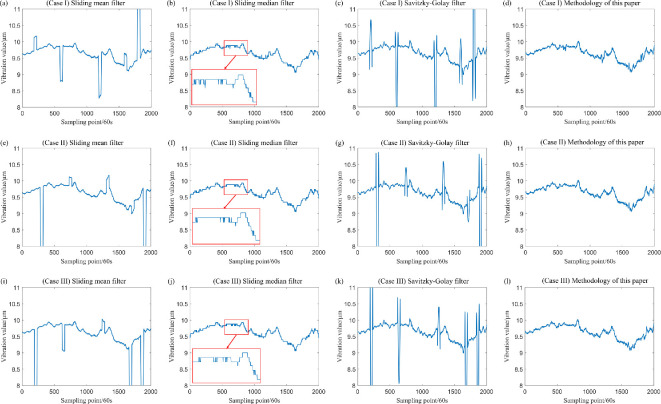
The drive-end shaft vibration data with added outliers were processed for comparative analysis using four different filtering methods.

**Figure 13.**
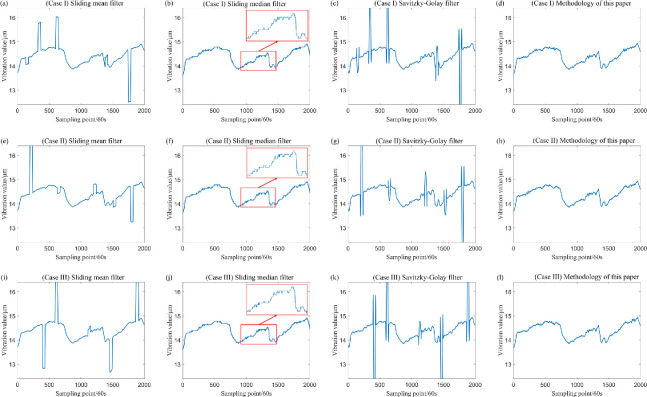
The exciter shaft vibration data with added outliers were processed for comparative analysis using four different filtering methods.

The data, after the inclusion of outliers, has been filtered, and the sliding window parameter has been established at k=20. In addition, the three aforementioned filtering methods have been employed for comparison with the filtering method proposed in this study, with the results illustrated in figures 12 and 13. A comparison of these figure reveals significant differences in the effectiveness of the four filtering methods when the measured sampling data contain outliers. Specifically, the classical sliding mean filter and the Savitzky–Golay filter exhibit noticeable deformation in the resulting curve near isolated and patchy outliers. Conversely, while the sliding median filtering method is not adversely affected by isolated or patchy outliers, it distorts the true trend of the data, leading to partial inaccuracies. By contrast, the filtering method proposed in this paper demonstrates minimal impact from outliers, effectively reflecting the genuine changes in the data. This outcome ensures both the reliability of the filtering process and the accuracy of the results.

**Figure 14.**
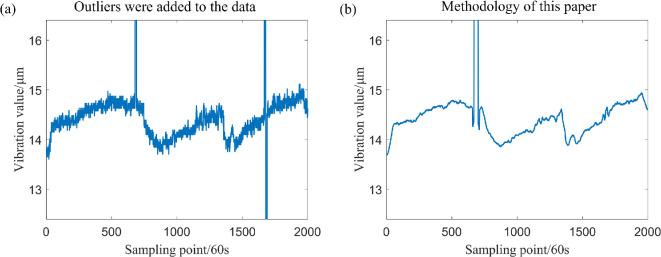
Illustration of sample breakdown points using exciter shaft vibration data after adding outliers.


To illustrate the sample breakdown point of the method presented in this paper more intuitively, we now analyse it through simulation experiments. Theoretically, when the number of outliers within the window does not exceed approximately 25% of the total data, these outliers will inevitably be positioned within an interval of about 25% on either the left or right after sorting. According to the constraints of the method proposed in this paper, it can be deduced that these outliers will not be included in the subsequent calculation process. Further analysis indicates that if the proportion of significantly large and small outliers in the window is approximately 25% each, then even if the total number of outliers in the window approaches 50%, the method employed in this paper still ensures that the resulting estimates remain unaffected by the outliers. For verification, we set the sliding window radius parameter k=20, which includes one patchy outlier with a patch length of 10 and two patchy outliers with distinctly large and small patch lengths of 9 within the window, as shown in equation (4.3). The simulation results are shown in figure 14.

(4.3) $$\begin{array}{rl} \left\{{\overset{˘}{y}}_{IV}^{2}(t_{i}\right) & =y(t_{i})+13|i=680,\ldots ,689\}\\ \left\{{\overset{˘}{y}}_{IV}^{2}(t_{i}\right) & =y(t_{i})+6|i=1670,\ldots ,1678\}\\ \left\{{\overset{˘}{y}}_{IV}^{2}(t_{i}\right) & =y(t_{i})-8|i=1680,\ldots ,1688\}. \end{array}$$

From the results of this simulation and the filtering outcomes on two types of compressor vibration data, it is evident that, with the sliding window radius parameter set to k=20, the method presented in this paper effectively mitigates the influence of patchy outliers when a patchy outlier of length 9 is present in the window data. In this case, the sample breakdown point approaches 1/4, as illustrated in figures 12 and 13. When two distinctly large and small patchy outliers are present within the window, the method similarly reduces the impact of these outliers, resulting in a sample breakdown point close to 1/2, as shown in figure 14. Conversely, when a patchy outlier of length 10 is present in the window data, it is observed that the extreme value significantly affects the filtering results, as shown in figure 14.

**Figure 15.**
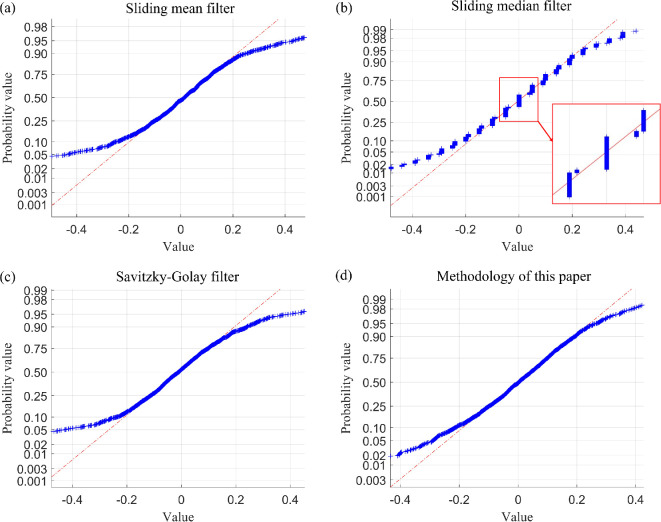
Normal probability plot of filtered residuals using four filtering methods for drive-end shaft vibration data after adding outliers (case I).

**Figure 16.**
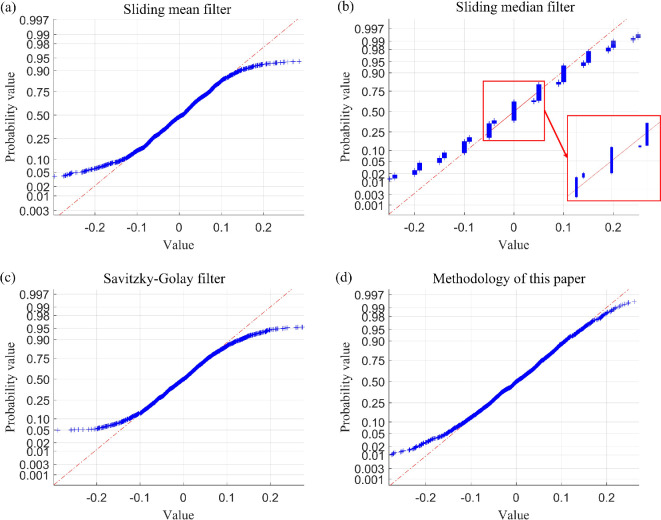
Normal probability plot of filtered residuals using four filtering methods for exciter shaft vibration data after adding outliers (case I).

The performance metrics of the aforementioned filtering methods, including sliding mean filtering, sliding median filtering, Savitzky–Golay filtering and sliding window constrained fault-tolerant filtering, have been analysed. Since the ability to filter random errors primarily depends on different filtering methods, to simplify the analysis process, this paper only selects one case (case I) for the analysis of random errors in filtering residuals. The results of this analysis are presented in tables 5 and 6 and figures 15 and 16.

**Table 5.** Performance evaluation of the drive-end shaft vibration data filtering effects.

filtering method	case I		case II		case III	
	DDCI	LFDC	DDCI	LFDC	DDCI	LFDC
sliding mean filter	0.1335	0.0421	0.1155	0.0418	0.1300	0.0447
sliding median filter	0.0041	0.0147	**0.0012**	0.0146	**0.0027**	0.0146
Savitzky–Golay filter	0.1662	0.0842	0.1461	0.0816	0.1672	0.0821
methodology of this paper	**0.0039**	**0.0097**	0.0091	**0.0087**	0.0136	**0.0087**

**Table 6.** Performance evaluation of the exciter shaft vibration data filtering effects.

filtering method	case I		case II		case III	
	DDCI	LFDC	DDCI	LFDC	DDCI	LFDC
sliding mean filter	0.1115	0.0336	0.0985	0.0354	0.1500	0.0444
sliding median filter	**0.0011**	0.0136	**0.0011**	0.0138	**0.0017**	0.0137
Savitzky–Golay filter	0.1398	0.0664	0.1248	0.0688	0.1905	0.0859
methodology of this paper	0.0048	**0.0060**	0.0027	**0.0058**	0.0041	**0.0060**

As evidenced by figures 12 and 13 and the locally enlarged normal probability plot of the filtered residuals in figures 15 and 16, the sample points of the filtered residuals obtained through sliding median filtering exhibit substantial deviation from the reference line. This indicates that the distribution of these sample points does not align closely with the reference line, suggesting that the residuals of the filtered data are not purely random errors. Consequently, this filtering process inadvertently removes components of the data that encapsulate genuine variations, resulting in data deformation. By contrast, the sample points derived from the filtering method proposed in this paper are predominantly distributed near the reference line.

Building upon the preliminary judgment of filtering residual distribution patterns using normal probability plots, the introduction of confidence interval analysis can enhance the accuracy of evaluating a filtering method’s capability to suppress random errors. By calculating the confidence interval (95% confidence interval) of the filtering residual samples based on the median absolute deviation (MAD), if the interval contains zero and is relatively narrow, the filtering residuals align with the assumption of random errors. Conversely, if the interval deviates from zero or is excessively wide, it indicates the potential presence of unfiltered systematic bias or non-random components in the residuals, demonstrating that the algorithm requires further optimization. Taking the analysis of vibration data with outliers from the drive-end shaft (case I) as an example, as shown in table 7, the 95% confidence intervals for the filtering residuals of the moving average filter, moving median filter and polynomial filtering methods all exhibit issues of deviation from zero or excessive width. Combined with the normal probability plot, this confirms the superior capability of the proposed method in filtering random errors when the data contain outliers.

**Table 7.** Confidence interval for filtering residuals of drive-end shaft vibration data with outliers (case I).

filtering method	confidence interval
sliding mean filter	[0.0064, 0.0204]
sliding median filter	[−0.0065, 0.0065]
Savitzky–Golay filter	[0.0032, 0.0170]
methodology of this paper	[−0.0037, 0.0092]

Combining the sample breakdown point analyses presented above, along with figures 3–16 and tables 1–7, we can conclude that the method proposed in this paper effectively extracts genuine changes in the measurement object from data series that are obscured by noise and contaminated by outliers. As discussed in the preceding section and illustrated in figures 3–16 and tables 1–7, the sliding median filter effectively mitigates the impact of isolated outlier and patchy outliers, and the sample breakdown point associated with this method is superior to that of the proposed approach. However, the local fluctuation difference metric of the sliding median filter’s curve is less robust than that of the method presented in this paper. If this metric is large, it indicates that the changes between adjacent data points in certain local areas significantly differ from the changes at other time points. This suggests that the curve exhibits noticeable local data distortion or non-smoothness. This deformation significantly influences the curve, more so than the proposed method. In the assessment of random errors within filtering residuals, the 95% confidence intervals for residuals obtained via the moving average filter, moving median filter and polynomial filtering methods consistently exhibit either deviation from zero or excessive interval width. Comprehensive evaluation incorporating normal probability plots further demonstrates that these conventional methods exhibit limitations in suppressing random errors when processing outlier-contaminated data. By contrast, the approach proposed in this study demonstrates markedly superior performance in random error suppression. Additionally, the curves generated by the proposed method are significantly less susceptible to deformation compared with those produced by the sliding mean filter and the Savitzky–Golay filter. This finding indicates that the data deformation resulting from outlier interference is markedly reduced when employing the proposed method, and the local fluctuation difference performance metric, as well as the ability to eliminate random errors, surpasses that of both the sliding mean filter and the Savitzky–Golay filtering technique. Therefore, the method presented in this paper demonstrates a more reliable fidelity tolerance capability when dealing with outliers in the data compared with sliding mean filtering, sliding median filtering and Savitzky–Golay filtering.

## 5. Conclusion

The data collected within the petrochemical industry are frequently subject to interference from various factors, including equipment noise, disrupted data transmission and sensor malfunctions. These issues lead to inconsistencies in data quality, the presence of noise pollution and the occurrence of isolated outliers or patchy outliers. Such complications have a direct impact on the accurate perception of operational conditions and the safety management of petrochemical production processes.

In this paper, we propose a sliding window constrained fault-tolerant filtering method for noise reduction that is based on quartiles. This method applies constrained processing to each data point within the window, effectively mitigating the influence of outliers and demonstrating robust fault tolerance. Consequently, it more reliably extracts meaningful non-random components of the signal. Additionally, we implement corresponding extensions at both ends of the data sequence to address the issue of unprocessed raw data at the boundaries of the sequence.

The sliding mean filter and Savitzky–Golay filter methods perform well when processing data without outliers. However, when confronted with outliers, they exhibit significant distortion, indicating poor fault tolerance. The sliding median filter method can effectively mitigate the impact of outliers. Nevertheless, this method filters out components in the data that hold practical significance, resulting in severe distortion and deformation of the filtering results, which interferes with the analysis of the true trend of data changes.

Experimental results demonstrate that when outliers are present in the data, compared with sliding mean filtering, sliding median filtering and Savitzky–Golay filtering, the method proposed in this paper can more effectively eliminate the influence of isolated outliers and patchy outliers, with its sample breakdown point being close to 1/4. At the same time, it can more reliably extract the non-random components in the data. The filtering results can better reflect the true trend of data changes, ensuring the accuracy of the filtering process and enhancing the reliability of the overall processing results. This achieves high-fidelity filtering results, providing a robust data processing technical support for the monitoring and optimal decision-making of petrochemical industrial processes.

## Data Availability

Data and relevant code for this research work are stored in GitHub [25] and have been archived within the Zenodo repository [26].
